# Identifying riparian climate corridors to inform climate adaptation planning

**DOI:** 10.1371/journal.pone.0205156

**Published:** 2018-11-14

**Authors:** Meade Krosby, David M. Theobald, Robert Norheim, Brad H. McRae

**Affiliations:** 1 Climate Impacts Group, College of the Environment, University of Washington, Seattle, Washington, United States of America; 2 Conservation Science Partners, Fort Collins, Colorado, United States of America; 3 The Nature Conservancy, Fort Collins, Colorado, United States of America; Universita degli Studi di Napoli Federico II, ITALY

## Abstract

Riparian habitats have been frequently identified as priority areas for conservation under climate change because they span climatic gradients and have cool, moist microclimates relative to surrounding areas. They are therefore expected to act as dispersal corridors for climate-induced species range shifts and to provide microclimatic refugia from warming. Despite recognition of these values, rigorous methods to identify which riparian areas are most likely to facilitate range shifts and provide refugia are currently lacking. We completed a novel analysis across the Pacific Northwest, USA, that identifies potential riparian corridors featuring characteristics expected to enhance their ability to facilitate range shifts and provide refugia. These features include large temperature gradients, high canopy cover, large relative width, low exposure to solar radiation, and low levels of human modification. These variables were used to calculate a riparian climate-corridor index using a multi-scale approach that incorporates results ranging in scale from local watersheds to the entire Pacific Northwest. Resulting index values for potential riparian corridors in the Pacific Northwest were highest within mountainous areas and lowest within relatively flat, lowland regions. We also calculated index values within ecoregions, to better identify high-value riparian climate corridors within the relatively flat, degraded areas where they may most contribute to climate adaptation. We found that high-value riparian climate-corridors are least protected in flat, lowland areas, suggesting that such corridors should be high priorities for future conservation effort. Our analysis provides critical information on valuable riparian climate-corridors to guide climate adaptation efforts (and riparian management and restoration efforts) in the Pacific Northwest, while offering a novel approach that may be applied to similar efforts in other geographies.

## Introduction

As climate change progresses and concern grows over the ability of species and ecosystems to adapt [1–2], considerable effort has been devoted to identifying areas on the landscape expected to promote biological resilience to change [3–5]. Riparian areas have been frequently identified as important features to conserve for climate adaptation [6–9], because they span the climatic gradients species are likely to follow as they track shifting areas of climatic suitability [10–12] and contain microclimates that are significantly cooler and more humid than immediately surrounding areas [13]. For these reasons, they are expected to provide dispersal corridors for species undergoing climate-induced range shifts [7,9] and microclimatic refugia from warming for species with limited movement capacities [14,5–6]. Riparian areas may also offer especially effective conservation umbrellas under climate change, because they disproportionately contribute to regional species richness [15–16], provide habitat for many upland species as well as riparian specialists [15–16], and directly contribute to the climate resilience of adjacent freshwater aquatic habitats [17–18]. Despite this recognition, few methods have been proposed for identifying priority riparian areas for climate adaptation.

Riparian areas are frequently prioritized in conservation planning efforts (e.g., [19–20]), but there are few examples of approaches aimed at identifying those that are most likely to promote climate adaptation. Available approaches for identifying riparian corridors to promote climate-induced range shifts include a conservation planning analysis for South Africa that included riparian corridors constructed by applying a fixed buffer around rivers connecting coastal to inland habitats to promote elevational species range shifts [21]. Similarly, riparian areas associated with 2^nd^ order streams linking the Pacific Ocean to high elevations were prioritized in a climate adaptation analysis for California, USA [22]. In another analysis, a land facet corridor analysis aimed at promoting species range shifts in Arizona, USA, connected large blocks of natural habitat using riparian corridors identified by applying a fixed buffer around expert-identified streams and riparian habitats [23]. Most of these analyses used rivers as coarse proxies for riparian habitat, and none rigorously accounted for variability in riparian area quality, which we argue strongly influences the degree to which riparian areas may facilitate range shifts and provide refugia.

To address the need for a rigorous approach to identify priority riparian areas for climate adaptation, we completed a novel analysis that identifies potential riparian corridors expected to promote the ability of biodiversity to respond to climate change. Specifically, we developed a riparian climate-corridor index to quantify the degree to which riparian areas may promote range shifts and provide refugia, identifying those riparian areas that: 1) span large temperature gradients, 2) have high levels of canopy cover, 3) are relatively wide, 4) have low solar insolation, and 5) exhibit low levels of human modification. These variables were derived from the theoretical and empirical literature on species’ responses to observed and projected climatic change. For example, riparian corridors that span large climatic gradients may help promote climate-induced range shifts from warmer to cooler areas [11–12]; riparian areas are already used as movement corridors for both riparian and upland species [24–26], and those spanning climatic gradients may offer particularly effective conduits for range migration, particularly across flat, degraded landscapes [27]. The effectiveness of such corridors would be further enhanced by high levels of canopy cover and greater riparian area width, features that have been shown to increase wildlife use of riparian areas as movement corridors [25], and to help moderate temperatures within riparian areas and promote the resilience of neighboring aquatic systems [17, 28]. Riparian corridors with lower exposure to solar insolation may also feature cooler temperatures and greater moisture [13, 29], increasing their value as microclimatic refugia [30–32]. Finally, riparian corridors with lower levels of human modification are likely to be more permeable to wildlife movement [33], while also being less vulnerable to exotic species invasion and other stressors that may inhibit species movements and reduce refugia quality [34].

Because these characteristics are likely to vary by the scale of analysis, and because scales of climate-induced range shifts and microclimatic refugia are likely to vary among species and over time [35,14], we developed a multi-scale approach to calculating riparian climate-corridor index values that incorporates results ranging in scale from local watersheds to the entire Pacific Northwest, USA. We also evaluated the protected status of riparian climate-corridors to help inform potential conservation action for maintaining riparian climate-corridor networks. Our analysis may thus provide critical information for guiding riparian management and climate adaptation efforts in the Pacific Northwest, while offering a novel approach that may be applied to similar efforts in other geographies.

## Materials and methods

### Study area

**Fig 1:**
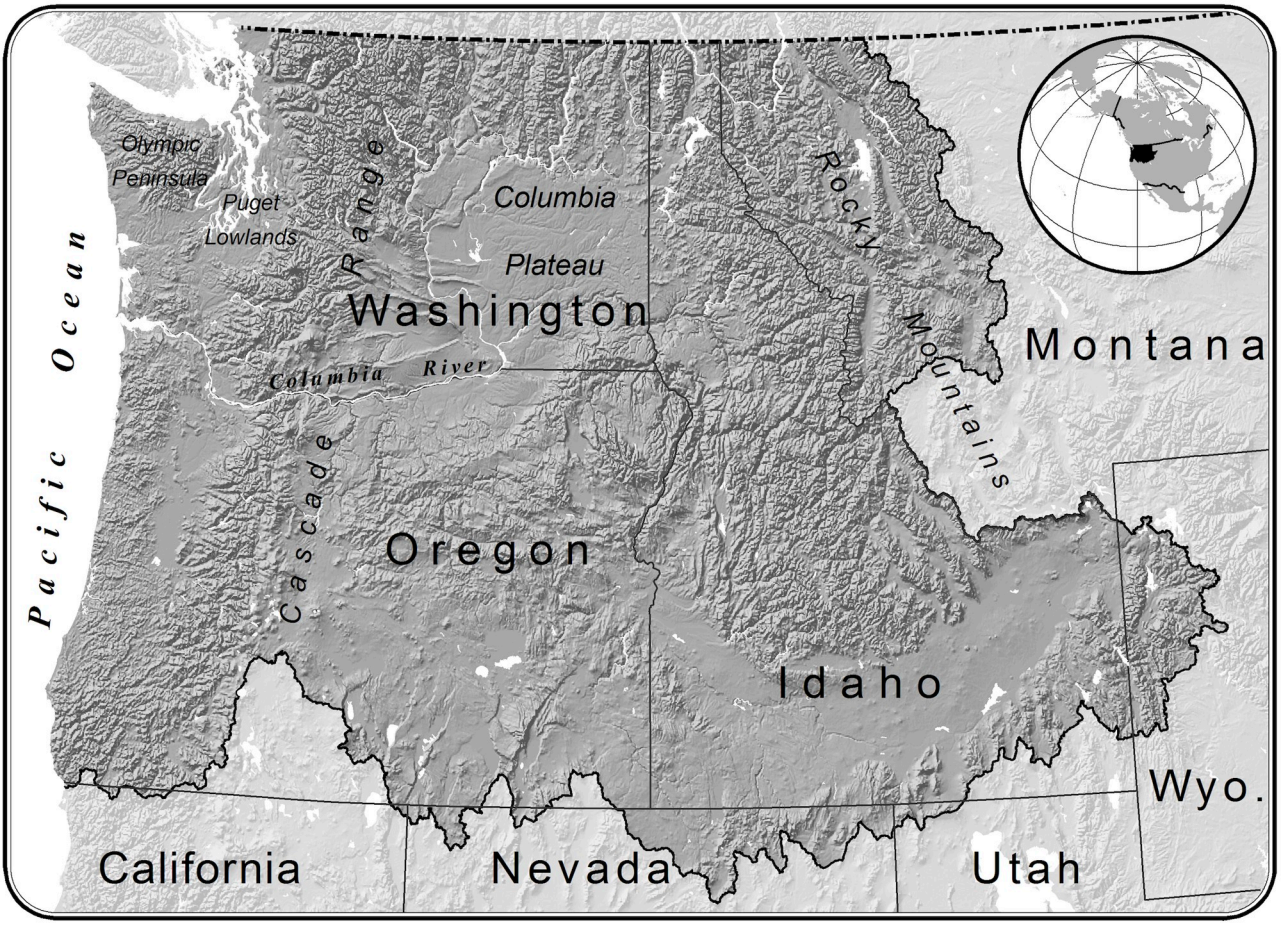
Analysis extent. We completed our analysis for the Pacific Northwest hydrologic region (Water Resource Region 17, in dark gray).

We completed our analysis for the Pacific Northwest, USA (USGS Water Resource Region 17; Fig 1). The Pacific Northwest includes a relatively cooler, moister region between the Pacific Coast and Cascade Range that is dominated by evergreen temperate forest; and a relatively drier region between the Cascade Range and Rocky Mountains that experiences more pronounced seasonality in temperature and features more diversity in vegetation types, from mixed forest at higher elevations to sagebrush-steppe in more arid lowlands.

### Analysis inputs

To identify high value riparian climate-corridors, we used a map of potential riparian areas identified by Theobald et al. [36], rather than a map of riparian vegetation. The potential riparian area map identifies the physical template where the dynamics of riparian vegetation are expected to occur, based on hydrological (stream discharge) and geomorphological (valley bottom shape) information rather than the (current) presence of riparian vegetation [36]. This 30 m data layer thus provides a comprehensive and consistent estimate of potential riparian area while avoiding many of the data gaps and inconsistencies [37] associated with existing maps of riparian vegetation derived from land cover (e.g., US LANDFIRE, US Fish & Wildlife Service National Wetland Inventory), which often have difficulty distinguishing riparian from non-riparian vegetation at 30 m resolution [37]. The potential riparian area dataset also provides key additional data layers (e.g., flow direction; see below) required by our analysis.

Our analysis aimed to identify the extent to which riparian corridors span large temperature gradients, have high levels of canopy cover, are relatively wide, have low exposure to solar radiation, and exhibit low levels of human modification. Our analysis thus included the following five variables (Table 1): mean annual temperature, canopy cover, riparian area width, potential relative radiation, and landscape condition.

**Table 1:** Analysis variables and source data.

Analysis Variable	Base Layer	Base Layer Resolution	Year Represented by Base Layer	Base Layer Sources
Mean Annual Temperature (*T*)	PRISM Mean Annual Temperature (downscaled using Climate WNA)	90 m	1961–1990 (mean historical temperature)	Daly et al. [39] (http://prism.oregonstate.edu/), Wang et al. [38] (http://climatewna.com/)
Canopy Cover (*C*)	NLCD Percent Canopy Cover	30 m (resampled to 90 m using bilinear interpolation)	2011	National Land Cover Dataset [40]
Riparian Area (*A*)	Potential Riparian Area	90 m	2009 (digital elevation model)	Theobald et al. [36]
Potential Relative Radiation (*R*)	Potential Relative Radiation (calculated using digital elevation model)	30 m (resampled to 90 m using bilinear interpolation)	2009 (digital elevation model)	This study, following methods of Pierce et al. [43], and using a digital elevation model from the National Elevation Dataset (http://ned.usgs.gov/).
Landscape Condition (*L*)	Landscape Condition	270 m (resampled to 90 m using bilinear interpolation)	2010 (roads); 2006 (development); 2001 and 2006 (landcover)	Western Association of Fish and Wildlife Agencies Crucial Habitat Assessment Tool [46], based on the NatureServe Landscape Condition Model [44]

We calculated mean annual temperature (*T*) as the 30-year mean of mean annual temperatures from 1961–1990, using a 90 m digital elevation model and the ClimateWNA tool [38], which extracts and downscales PRISM [39] monthly data and calculates climate variables for specific locations based on latitude, longitude, and elevation. For canopy cover (*C*), we used the percent tree canopy cover dataset for 2011 from the National Land Cover Dataset [40, 41]. We calculated potential riparian area (*A*), a measure of the width of potential riparian areas, directly from the 30 m potential riparian area data layer from Theobald et al. [36]. We used the 30 m National Elevation Dataset [42] to calculate potential relative radiation (*R*), a unitless measure of solar radiation that takes into account temporal changes in solar orientation as well as topographic shading from adjacent landforms [43]; such shading has been shown to contribute to lower temperatures in complex terrain [13–14]. We used the landscape condition (*L*) model [44] as a measure of the degree to which potential riparian areas have been affected by human activities. Although a more recent and higher-resolution dataset on human modification was available [45], we used *L* to be consistent with the Western Association of Fish and Wildlife Agencies Crucial Habitat Assessment Tool [46].

### Calculating a riparian climate-corridor index

We calculated an index of riparian climate-corridor quality for individual, ecologically-relevant spatial units that we call “potential riparian corridors,” which we define as the potential riparian area that runs longitudinally along a stream/river from the stream outlet (or mouth) up through the hydrologic network of a watershed, ending at the stream initiation point (or headwater). For each potential riparian corridor, we calculated a riparian climate-corridor index using three main steps.

**Fig 2:**
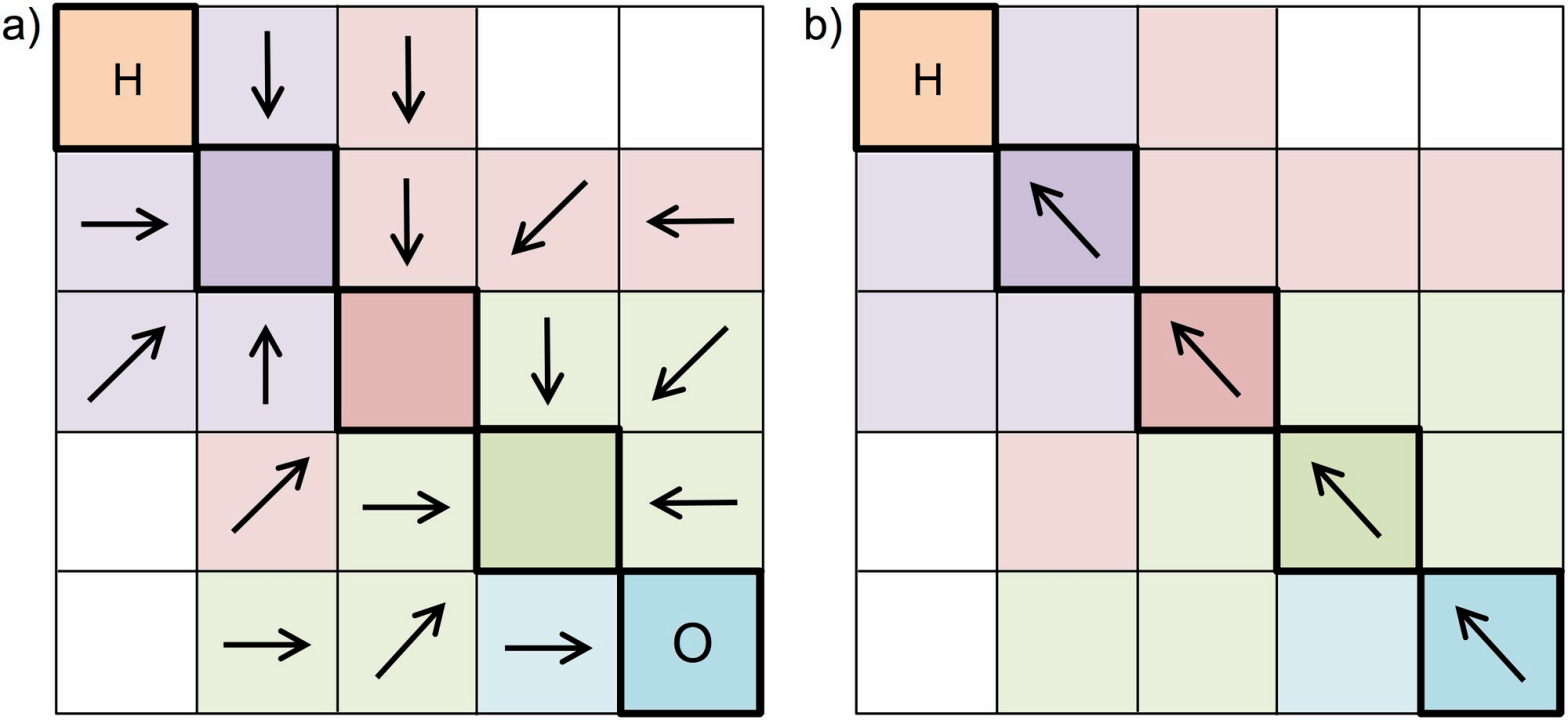
Using Flow Direction, Flow Accumulation, Flow Length tools to calculate the riparian climate-corridor index for a potential riparian corridor. a) For all potential riparian cells draining into a given cell in the streamline (outlined in bold, with it and all cells draining into it shown in the same color), we calculate, for each variable (*C*, *R*, *L*), the average value across the cells that contribute (accumulate) to the stream flow cell, and attribute this average value to the streamline cell (or the midline cell, for larger water bodies) using the Flow Direction and Flow Accumulation tools in ArcGIS. We calculate *A* as the number of potential riparian cells draining into the streamline cell. b) We then calculate, for each variable (*C*, *R*, *L*, *A*), the average value across all streamline cells from the outlet (O) to the headwater (H), and attribute this average value to the headwater using the Flow Length tool in ArcGIS. We also attribute a value for *T*, calculated as the absolute difference in temperature between the outlet and headwater cells. We then standardize (0–1) the average value for each variable (for equal weighting) and calculate the index, attributing this index value to the headwater cell. This is repeated for each downstream outlet until reaching the ocean, each time attributing the index value to the headwater cell. Each streamline cell is then given the average index value attributed to its upstream headwaters.

First, we accumulated the values of four variables (*C*, *A*, *R*, *L*) from locations (cells) within potential riparian areas laterally (i.e., orthogonal to the neighboring stream) to the nearest cell along the central flow path that follows the mid-line of streams/rivers (Fig 2a).

**Fig 3:**
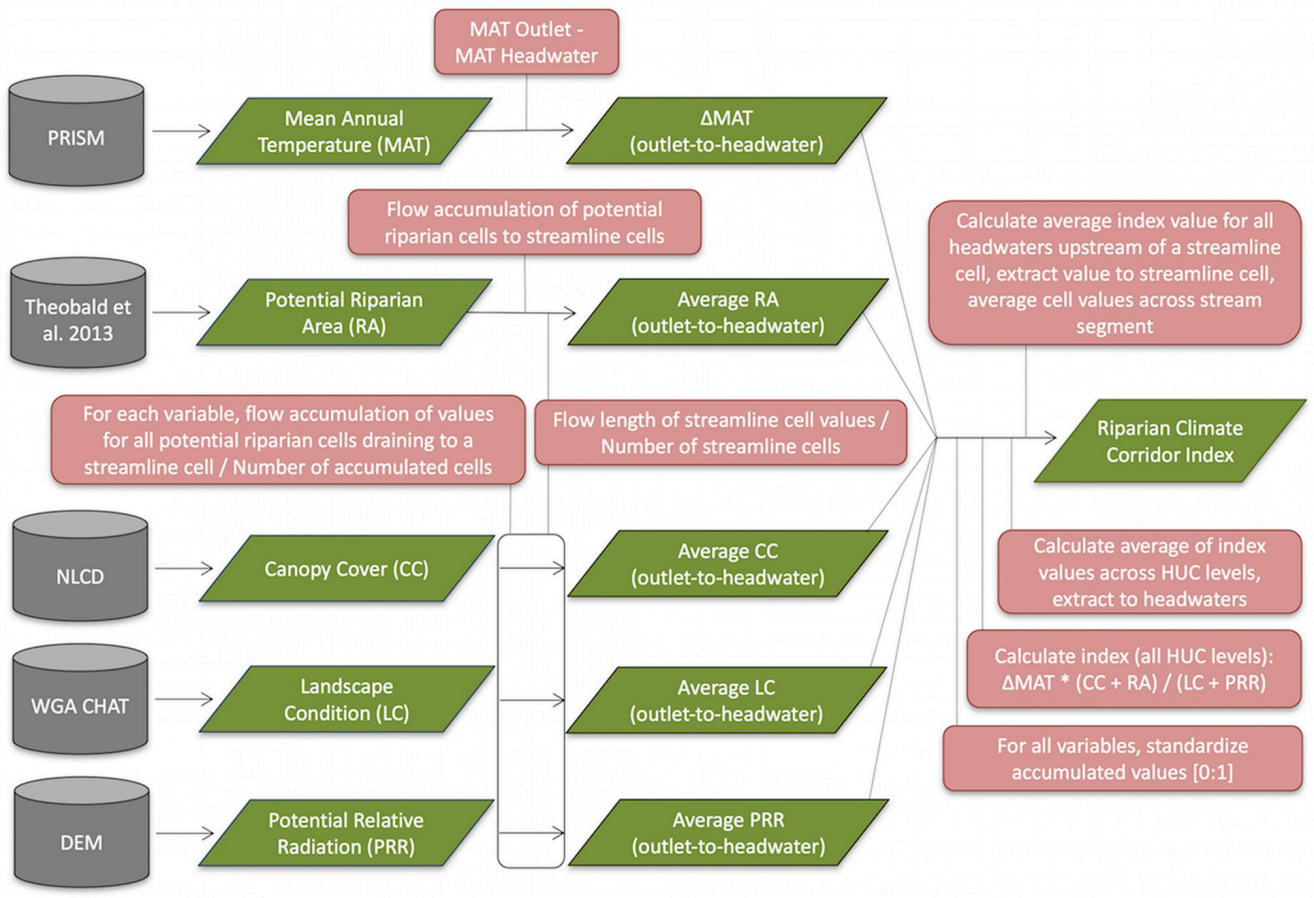
Summary of modeling approach, including key inputs, outputs, and analysis steps. Data sources are shown in gray, processing steps in pink, and inputs and outputs in green.

Second, we accumulated the values longitudinally along the central flow path within the stream/river, from its outlet to its headwater (Fig 2b). We accumulated values upstream rather than downstream to simulate the process of upward range movement along riparian corridors, from watershed outlets toward higher-elevation headwaters. Accumulating upstream also allowed us to calculate index values for individual riparian corridors adjacent to a stream/river reach running between its headwater and watershed outlet, because accumulating downstream would result in a single accumulated index value for an entire watershed. Third, we used these accumulated variable values to calculate an index of climate adaptation quality for the riparian climate-corridor from the outlet to headwater. Representing potential riparian corridors using a raster representation (rather than stream line vectors) allowed us to account for subtle gradients and variations within potential riparian areas—vital information lacking in previous studies. For rivers wider than 90 m, we excluded water cells when calculating variable values. Note that we represented spatial features, such as elevation and land cover, at 30 m resolution, but accumulated the up-scaled data at 90 m for computational purposes. A more detailed description of our analysis is provided below, and summarized in Fig 3.

#### 1. Accumulate values within potential riparian corridors laterally to the stream line

We clipped the *C*, *R*, and *L* rasters to the extent of the potential riparian area. We then accumulated C, R, and L values along flow paths for all potential riparian cells draining into a given cell that represents the center of the stream line (i.e. the central flow path). That is, each cell located along the central flow path was attributed with the sum of the values for that variable for all the potential riparian cells that drain into it, using hydrologic tools in ArcGIS v10 software [47]. We then divided the accumulated value for each variable by the number of accumulated cells, so that, for each variable, each central flow path cell in the adjacent stream/river was ultimately attributed with the average variable value for its contributing potential riparian cells. The flow-accumulated area for the potential riparian area (*A*) was calculated in a similar manner, by accumulating the number of potential riparian cells draining into each central flow path cell in the adjacent stream/river. In cases where no potential riparian area cells drained into a central flow path cell, values for each variable were measured for only the central flow path cell itself, which was given an *A* value of 1.

#### 2. Accumulate values for each variable longitudinally from stream/river outlet to headwater

We accumulated values along individual streamlines running from the watershed outlet to a stream’s headwater, for each of the four variables (*C*, *A*, *R*, *L*). These accumulated values were then extracted to the central flow path cell at the stream/river’s headwater, and divided by the number of contributing central flow path cells, to provide an average value for each variable for the associated potential riparian corridor. Mean annual temperature (*T*) was also extracted at each watershed outlet (or sink, in the case of closed basins) and for each headwater, and the difference between the two calculated and extracted to each stream/river’s headwater. The average value for each variable was then divided by the largest value for that variable within the full study region, to standardize values to the range 0:1.

#### 3. Calculate riparian climate-corridor index for each watershed-scale riparian corridor

We used the averaged, standardized values for each variable to calculate a Riparian Climate-Corridor Index for each watershed-scale riparian corridor, using the following formula:

RiparianClimate-CorridorIndex=ΔT×[(C+A)/(R+L)]

Index values will thus be highest for those riparian corridors with the largest change in temperature (*T*) from outlet to headwater, highest percent canopy cover (*C*), greatest width (*A*), lowest exposure to solar radiation (*R*), and lowest level of human modification (*L*). Where *ΔT* was negative (indicating a higher temperature at the headwater than at the outlet), the index value was set to 0, to maintain higher index values for corridors leading from warmer to cooler areas across scales (see description of multi-scale approach, below). Our analysis is thus similar to other climate-gradient corridor approaches [11–12] in that it prioritizes corridors connecting warm areas to cool (in this case, headwater and outlets) using pathways that follow monotonic gradients (i.e., moving along gradients in only one direction, from warm to cool). All index values were extracted to the headwater associated with each potential riparian corridor.

#### 4. Account for scale effects

We calculated a multi-scale, riparian climate-corridor index using the above procedure for riparian corridors within 6^th^, 5^th^, 4^th^, 3^rd^, 2^nd^, and 1^st^ field HUCs (i.e., nested watersheds, from smallest to largest, respectively). HUCs are hierarchical hydrologic unit codes (HUC) assigned to all watersheds in the US [48]; the watershed cataloguing system nests watersheds into progressively larger units, similar to Pfafstetter codes that are also used globally. Our method should thus be applicable to any similar watershed cataloguing system in other countries. This procedure resulted in up to six index values being extracted to each headwater, corresponding to the index values of progressively longer downstream potential riparian corridors adjacent to each stream/river from its headwater to its outlet for progressively larger watersheds, eventually terminating at the ocean (or sink, in the case of closed basins). We scaled each of these nested index values to the range (0:1) and averaged them (equally-weighted), so that the final index value extracted to each headwater would reflect the climate adaptation value of all of its downstream riparian corridors. Finally, we calculated, for each individual central flow path cell within streams/rivers, the average of the index values attributed to all of its upstream headwaters. The final index values for each flow path cell within streams/rivers thus reflect the degree to which its adjacent potential riparian area cells are expected to help facilitate range shifts and provide refugia, from local to regional scales.

We also calculated a measure of riparian climate-corridor quality for entire watersheds by calculating the average of index values for all riparian climate-corridors within a given HUC. To account for differences in index values among ecoregions, and to more easily identify the highest quality riparian climate-corridors within each ecoregion, we binned all index values into 5 equal-area quintiles within each Level III ecoregion [49].

### GAP analysis and sensitivity testing

We evaluated the degree to which high-value riparian climate-corridors identified by our analysis fall within currently designated protected areas by measuring the GAP status of riparian climate-corridors within 1) the top quintile of index scores, 2) the top two quintiles, and 3) all quintiles, for both the entire Pacific Northwest and within ecoregions. GAP status codes are provided by the US Geological Survey’s Gap Analysis Program (GAP), and measure the degree to which lands in the US are managed for conservation [50]. Code 1 and 2 denote the highest degree of management for conservation (and meet the IUCN definition of protected), while Code 3 is given to lands that support multiple uses, including resource extraction. Code 4 lands are unprotected or have unknown management intent.

We also tested the sensitivity of the riparian climate-corridor index to the inclusion of individual input variables by removing individual variables one at a time, re-calculating the index, and measuring resulting differences across the study area. We also calculated correlation coefficients among these index values, as well as correlation coefficients among individual variables, to aid in interpretation of results.

## Results

### Riparian climate-corridor index values

**Fig 4:**
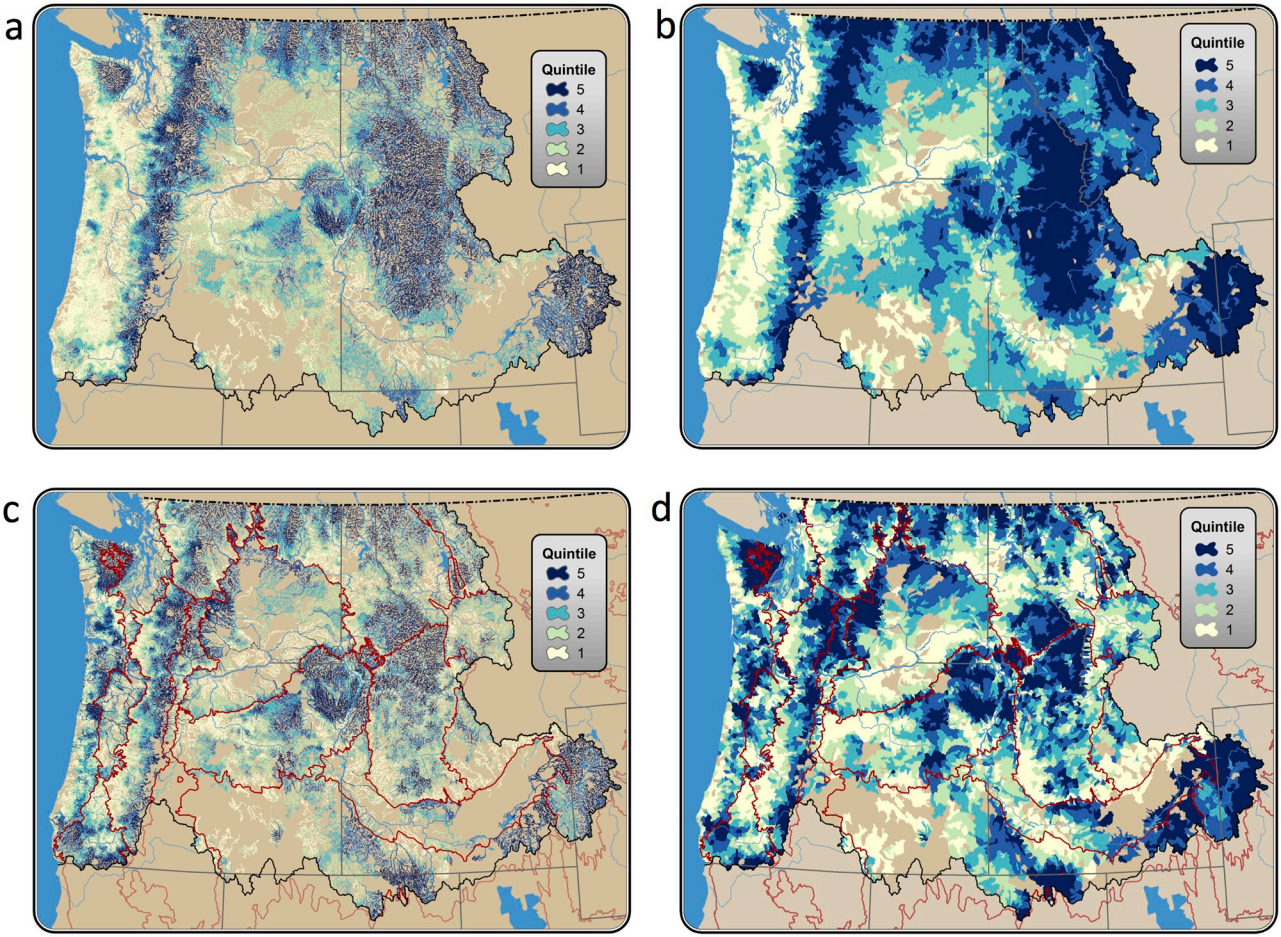
Riparian climate-corridor index values for the Pacific Northwest. Values are averaged across nested watershed scales (6^th^ to 1^st^ field HUCs), attributed to streamlines associated with potential riparian corridors. Values are shown by quintile for the Pacific Northwest, USA (panels a and b), and within ecoregions (panels c and d); and for both individual riparian corridors (panels a and c) and averaged across 6^th^ field HUCs (panels b and d).

**Fig 5:**
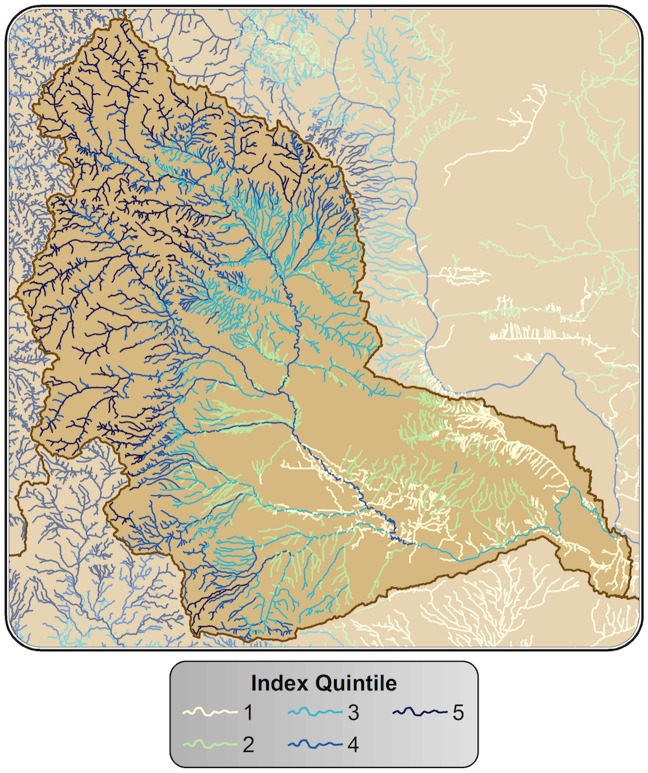
Riparian climate-corridor index values shown for an individual watershed. Values are shown by quintile and attributed to streamlines associated with potential riparian corridors.

**Fig 6:**
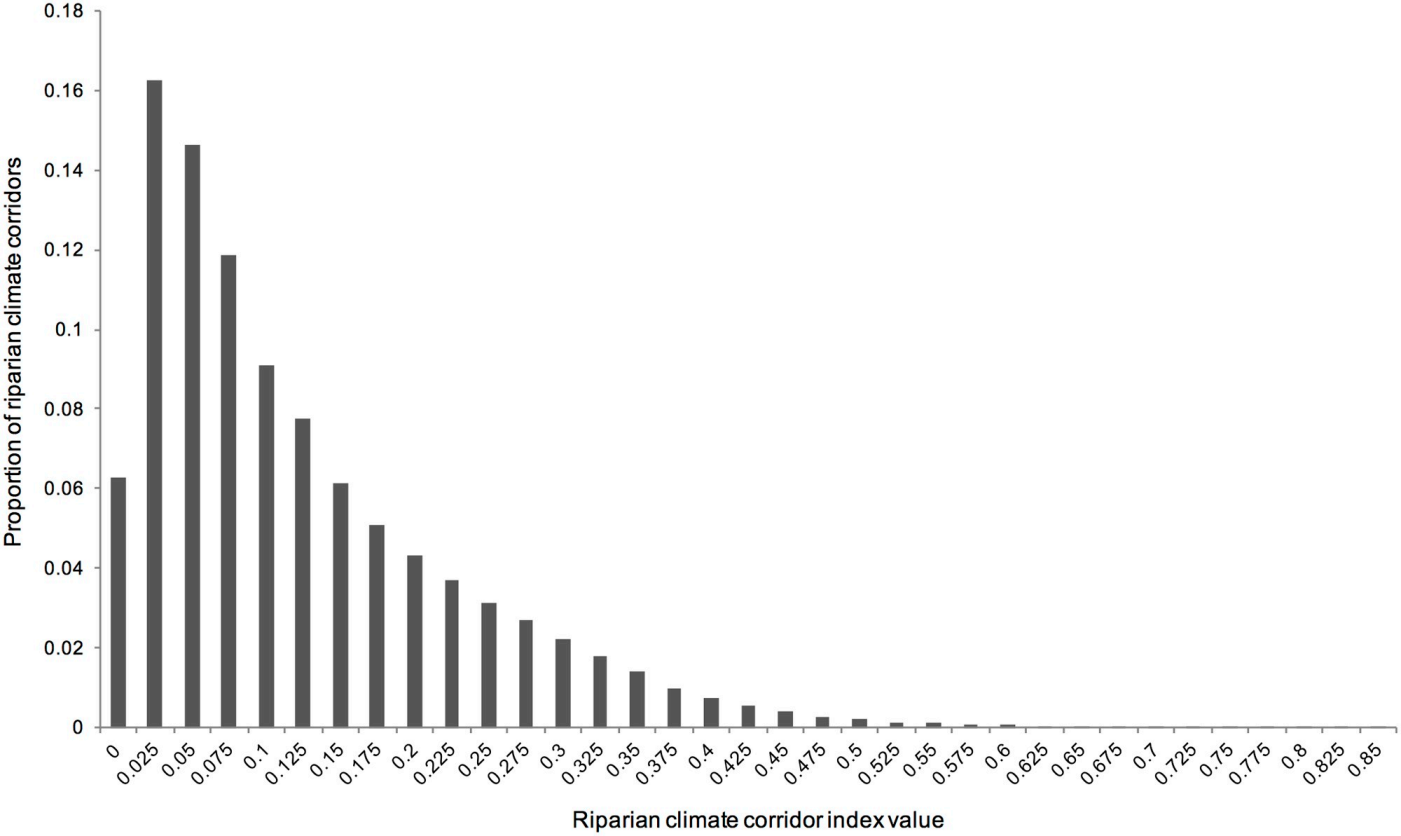
Distribution of riparian climate-corridor index values. Shown for all watershed-scale riparian corridors in the Pacific Northwest.

**Fig 7:**
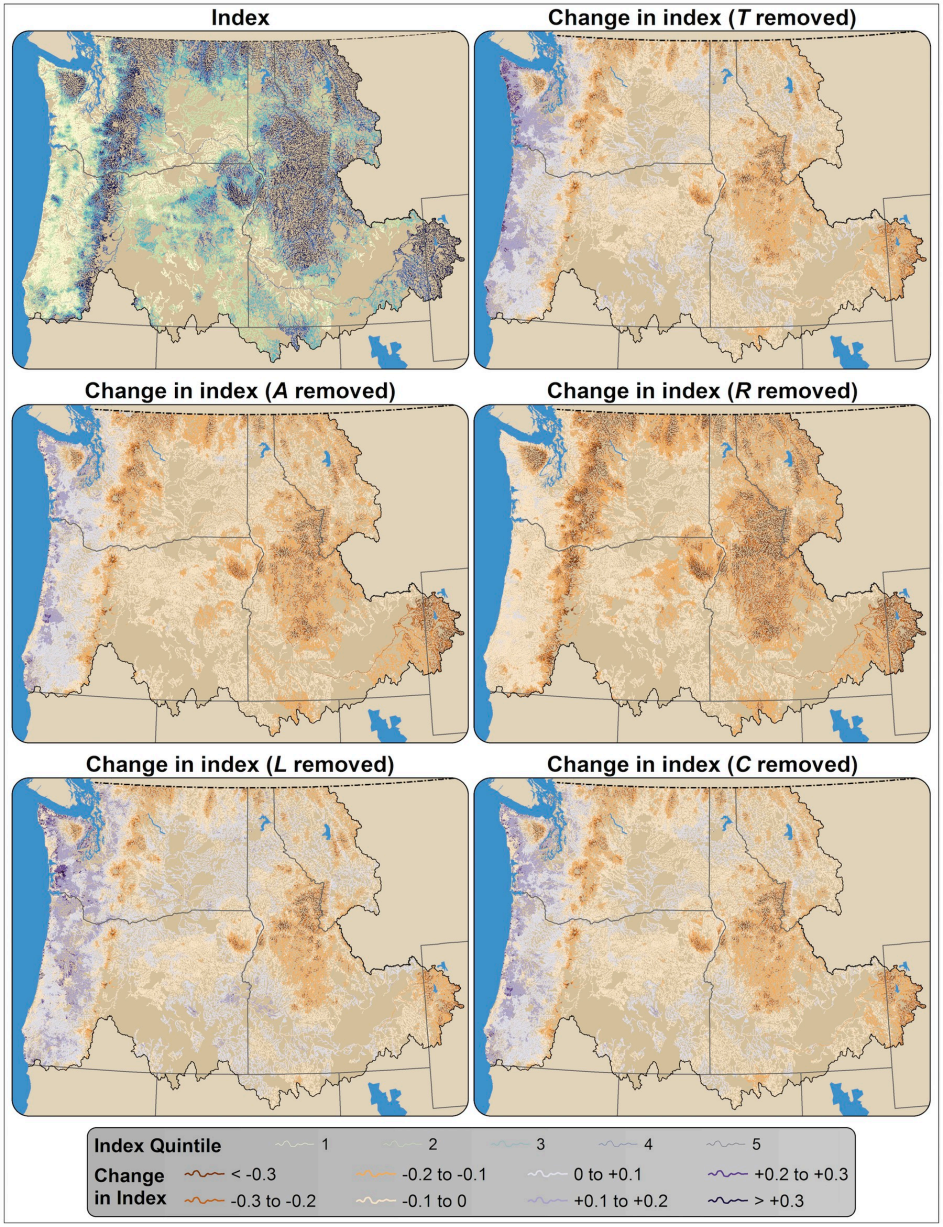
Sensitivity of riparian climate-corridor index values to individual analysis variables. Panels show the index with all variables included (top left), and the index with change in mean annual temperature (*T*) removed, with riparian area (*A*) removed, with potential relative radiation (*R*) removed, with landscape condition (*L*) removed, and with canopy cover (*C*) removed.

We found that the climate adaptation potential of riparian corridors varies considerably, both across the Pacific Northwest (Fig 4) and within individual watersheds (Fig 5). Index values ranged from 0 to 0.83 (Fig 6), with the highest index values found in mountainous areas (e.g., the Cascade Range), and the lowest index values found in relatively flat, lowland regions such as the Columbia Plateau. Mountainous areas exhibited higher *ΔT* scores, on average, as well as higher canopy cover (*C*), solar insolation (*R*), and landscape condition (S1–S4 Figs). These effects were amplified by positive correlations among all input variables but riparian area (S5 Fig): relatively flat areas with low *ΔT* tended to also have lower canopy cover (*C*), were in poorer landscape condition (*L*), and had higher solar insolation (*R*). Indeed, removing *ΔT* from the index calculation resulted in a spatial pattern similar to that seen when the calculation included *ΔT* (Fig 7); including *ΔT* generally reinforced the pattern of lower values in areas with gentler topographic relief (often near outlets) and higher values in mountains (often near headwaters).

Most potential riparian corridors had relatively low index values (Fig 6). The relatively high number of potential riparian corridors with index values equal to 0 is due in large part to the relatively cool temperatures of the Pacific Northwest coast; many interior headwaters have warmer mean annual temperatures than their streams’ coastal outlets. Because negative *ΔT* values were converted to zero and *ΔT* is multiplied by the rest of the index, such potential riparian corridors receive a zero value, though they may otherwise be of high quality (Fig 7). For example, the low index scores received by otherwise high-quality riparian areas in the western Olympic Peninsula were due to negative or relatively low *ΔT* between coastal stream outlets and headwaters (Fig 7, S1 Fig).

Areas with no headwaters (and thus no index scores) were seen in regions lacking surface water due to high aridity and/or high soil permeability (Fig 4).

### GAP analysis and sensitivity testing

**Fig 8:**
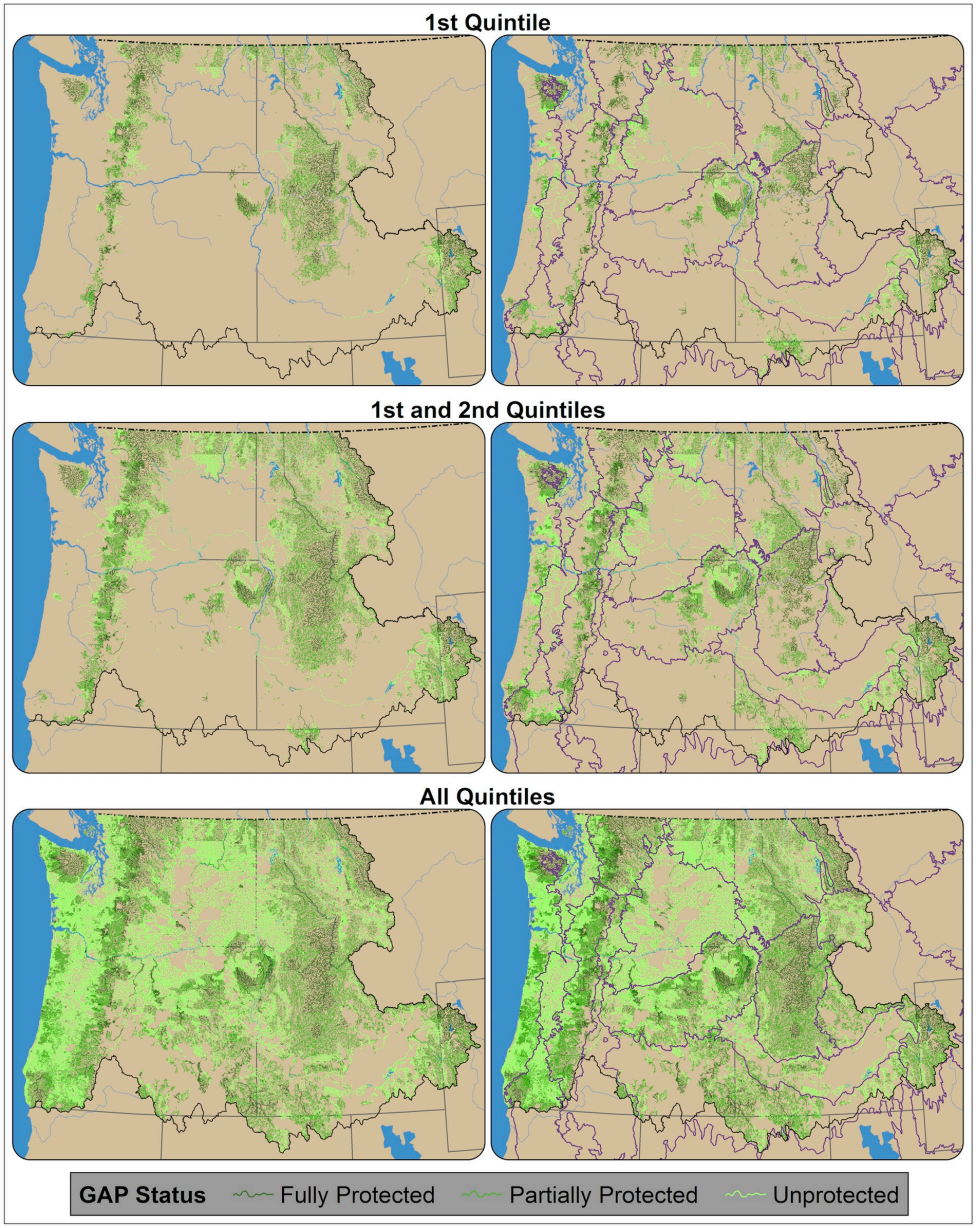
GAP status of riparian climate-corridors. GAP status is shown for riparian corridors within the top quintile of riparian climate-corridor index values (top row), top two quintiles (middle row), and all quintiles (bottom row); by both the entire Pacific Northwest (left column) and within ecoregions (right column). GAP status is shown for fully protected (GAP status 1 and 2; forest green), partially protected (GAP status 3; kelly green), and unprotected (GAP status 4; lime green) riparian climate-corridors.

We found that riparian climate-corridors varied regionally in their level of protection (Fig 8). For riparian climate-corridors with the highest 20% of index scores, 35.5% were fully protected (GAP status 1–2) and 50.4% were partially protected (GAP status 3) across the Pacific Northwest. Within ecoregions, GAP status of riparian climate-corridors with the highest 20% of index scores varied from 83.8% fully protected and 14.6% partially protected in the North Cascades, to 1.3% fully protected and 18.8% partially protected in the Columbia Plateau.

We found that riparian climate-corridor index values were relatively insensitive to individual input variables (Fig 7). Removal of individual variables from the index calculation resulted in little change to index scores across the study area, resulting in an average change in index values of -0.0126 for removal of mean annual temperature (*ΔT*), +0.0051 for landscape condition (L), -0.0168 for canopy cover (*C*), -0.0607 for riparian area (*A*), and -0.1154 for potential relative radiation (*R*). Given the strong correlations among index variable values and elevation (i.e., that relatively flat areas with low *ΔT* also have lower canopy cover (*C*) and landscape condition (*L*), and higher solar insolation (*R*)), variable exclusion generally resulted in decreased values in mountainous areas and increased values in lower-elevation areas. Exclusion of *R* had a slightly stronger effect on index values in mountainous areas (lowering index values), and exclusion of *T*, *C*, and *L* had a slightly stronger effect on lower-elevation coastal areas (increasing values).

## Discussion

Our analysis identified potential riparian corridors that span climatic gradients, have high canopy cover, low levels of solar exposure, low levels of human modification, and are relatively wide—characteristics expected to facilitate climate-induced range shifts and provide micro-climatic refugia. Not surprisingly, we found that potential riparian corridors in mountainous regions—which tend to be steep, forested, topographically shaded, and have low levels of human modification—had the highest riparian climate-corridor index values. We also found that potential riparian corridors in lowland areas—which tend to be flat and have low canopy cover, less topographic shading, and high levels of human modification—had the lowest values (Fig 4a and 4b). Because of the correlations of temperature with other variables, change in temperature—which we had expected to be a key variable for identifying riparian corridors with strong climatic gradients—in fact had a relatively modest impact on index scores (Fig 7), generally reinforcing the pattern of lower index values in areas with gentler topographic relief and higher values in mountains. The index is thus robust to our coarse approach to measuring temperature gradients along riparian corridors.

We also found that relatively flat and highly modified ecoregions (e.g., the Columbia Plateau and Puget Lowlands ecoregions; Fig 4c and 4d) had the least protected high-scoring riparian climate-corridors among Pacific Northwest ecoregions (Fig 8). High-scoring riparian climate-corridors in these areas thus suggest immediate priorities for conservation action (e.g., protection or restoration), as they may provide some of the best adaptation opportunities in flat, highly modified landscapes that may limit species range movements and persistence in microclimatic refugia. We also found that a large number of otherwise high-quality potential riparian corridors along the coast received low index scores, because their interior headwaters have warmer mean annual temperatures than their streams’ cooler, coastal outlets. These results emphasize that our index is designed to identify riparian climate-corridors expected to promote species range shifts from warmer to cooler areas, which may in some cases result in low scores for corridors that have high conservation value under static or current climates.

Index values for riparian climate-corridors along large rivers (e.g., the Columbia River) often had higher values than corridors within nearby lower-order streams (e.g., headwater streams). This is because higher-order streams frequently have tributaries at higher elevations; riparian climate-corridors associated with these higher-elevation tributaries tend to have relatively high index values, and the index values of riparian climate corridors along higher-order streams incorporate these upstream values. The high index values of riparian climate-corridors along higher order streams thus reflect their connectivity to high-scoring upstream corridors, and thus their capacity to promote range shifts and provide access to climatic refugia at a regional scale. Indeed, shorter riparian corridors, such as those that would be found along headwater streams, have been shown to be more effective at promoting species movements [51]. Thus, the trade-off of this multi-scale approach—designed to accommodate diverse species with needs for movement and refugia at a range of scales—is its potential to overlook riparian climate-corridors that may be valuable at a more local scale, but do not meaningfully contribute to broader-scale, regional adaptation. Identifying riparian climate-corridors with high index values within ecoregions (Fig 4c and 4d) or local watersheds (S6–S10 Figs) may help address needs for more local-scale prioritization.

We recommend considering several caveats when applying the riparian climate-corridor index. First, this approach only indirectly accounts for connectivity along riparian corridors; while index values will decrease with increasing human modification along a corridor, the effect of local but severe movement barriers (e.g., towns, cliffs) on index values could be muted if human modification is low elsewhere along the corridor, particularly at broader scales. The analysis could thus be improved by incorporating explicit connectivity measures that sufficiently penalize high-resistance, local barriers that could sever connectivity; a range of connectivity modeling approaches could be adapted for this purpose (e.g., [52]). The analysis could also be improved by further validating analysis inputs and assumptions, such as empirically measuring canopy cover and solar insolation across riparian areas and testing their influence on temperature, and, ultimately, range shifts and refugia. Future comparison of our index to other indices of riparian quality (e.g., [53]) would also aid in interpretation of results. Thus, we recommend using this analysis as a means of identifying priority riparian areas for additional evaluation (e.g., field validation, comparison with other data sets, integration with other conservation values) before making decisions regarding conservation action.

We also recognize the scaling challenges in mapping riparian vegetation and modeling potential riparian areas. Our analysis provides estimates of potential riparian climate-corridors at (>90 m) due to data resolution and computation limitations. Future work can apply our approach using high resolution data that have (or will likely) become available. An additional caveat is the risk of unintended negative consequences (e.g., spread of invasive species or disease) by protecting or restoring riparian climate corridors to promote species movements. Our analysis reduces this risk by prioritizing those riparian areas that are in good condition, and therefore expected to be less vulnerable to invasion. Further, previous research has shown that the benefits of corridors outweigh potential negative effects [54], including potential risks related to climate-induced range shifts [55]. Indeed, the synergistic threats of habitat loss, fragmentation and climate change present an urgent need to restore landscape features such as riparian corridors that have historically provided natural conduits for species movement.

Although riparian areas are expected to provide critical movement corridors and refugia under climate change [6–7,9], they are also among the most threatened habitats in many regions [56]. Our analysis offers a first step toward identifying, for large regions, those riparian areas most likely to promote species’ ability to respond to climate change, as well as those that may be most vulnerable to climate change and in need of restoration measures. Such information may offer valuable guidance for future investments in riparian protection and restoration as part of climate adaptation efforts.

## Supporting information

S1 Fig Mean annual temperature (*T*), based on the 30-year mean of mean annual temperatures from 1961–1990, using a 90 m digital elevation model and the ClimateWNA tool [34], which extracts and downscales PRISM [35] monthly data and calculates climate variables for specific locations based on latitude, longitude, and elevation. (TIFF)

S2 Fig Canopy cover (*C*), based on the percent tree canopy cover dataset from the National Land Cover Dataset [36]. (TIFF)

S3 Fig Potential relative radiation (*R*), a unit-less measure of solar radiation that takes into account temporal changes in solar orientation as well as shading effects from neighboring topography [38], based on a 30 m digital elevation model from the National Elevation Dataset [36]. (TIFF)

S4 Fig Landscape condition (*L*), provided by the Western Governors’ Association’s Crucial Habitat Assessment Tool (WGA 2013) as a measure of the degree to which potential riparian areas have been affected by human activities using the landscape condition model [39], where higher values correspond to lower landscape intactness. (TIFF)

S5 Fig Riparian area (*A*), based on the potential riparian area data layer from Theobald et al [32]. (TIFF)

S6 Fig Riparian climate-corridor index values averaged across individual watersheds (6^th^ field HUCs). (TIFF)

S7 Fig Riparian climate-corridor index values averaged across individual watersheds (5^th^ field HUCs). (TIFF)

S8 Fig Riparian climate-corridor index values averaged across individual watersheds (4^th^ field HUCs). (TIFF)

S9 Fig Riparian climate-corridor index values averaged across individual watersheds (3^th^ field HUCs). (TIFF)

S10 Fig Riparian climate-corridor index values averaged across individual watersheds (2^nd^ field HUCs). (TIFF)
