# Supplementary figures and images for: Identifying riparian climate corridors to inform climate adaptation planning
Source: PLoS One. 2018 Nov 14;13(11):e0205156. doi: 10.1371/journal.pone.0205156 (PMC6235256; doi:10.1371/journal.pone.0205156)

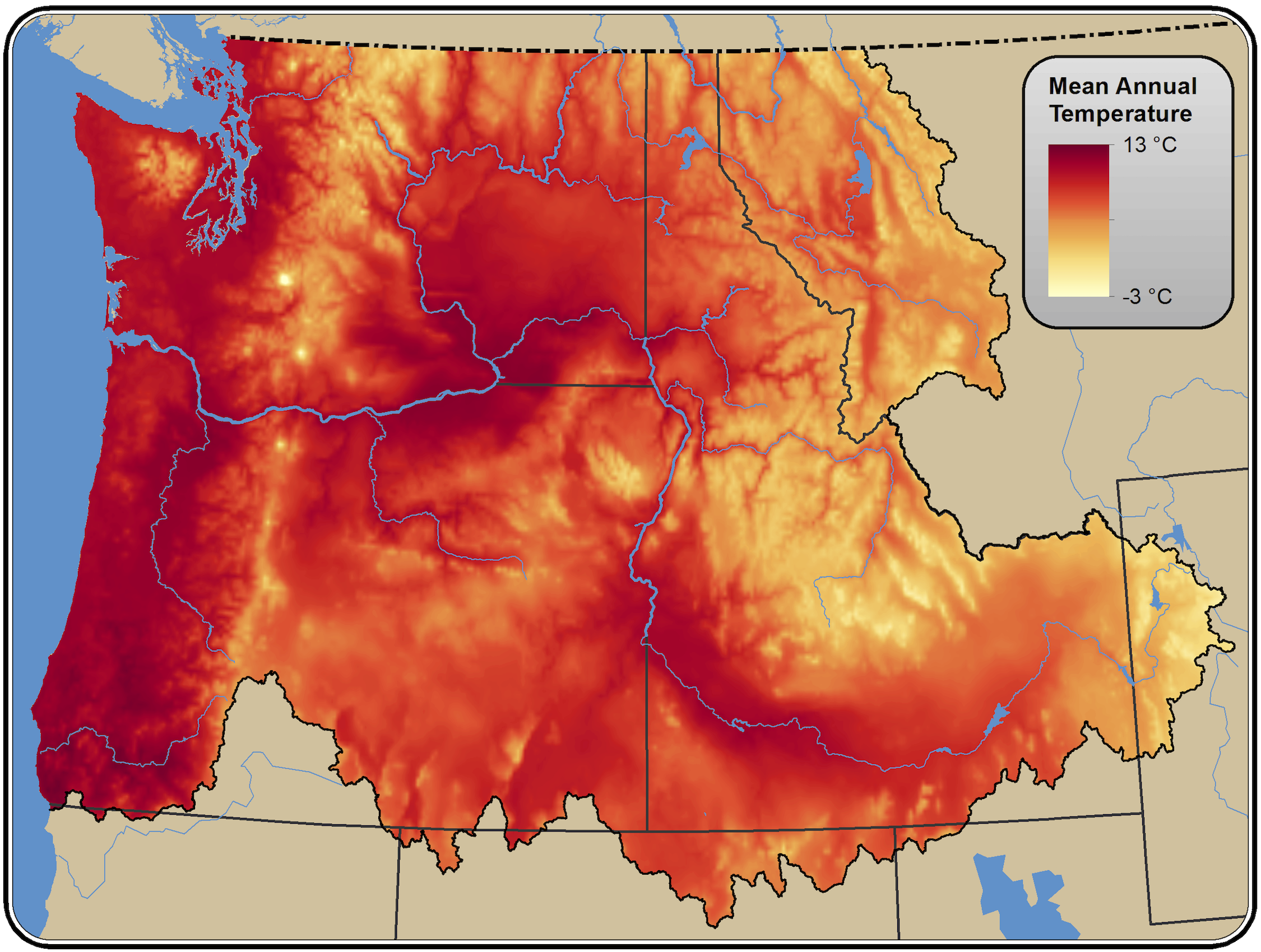

Supplement: S1 Fig — (TIFF) [file pone.0205156.s001.tiff]

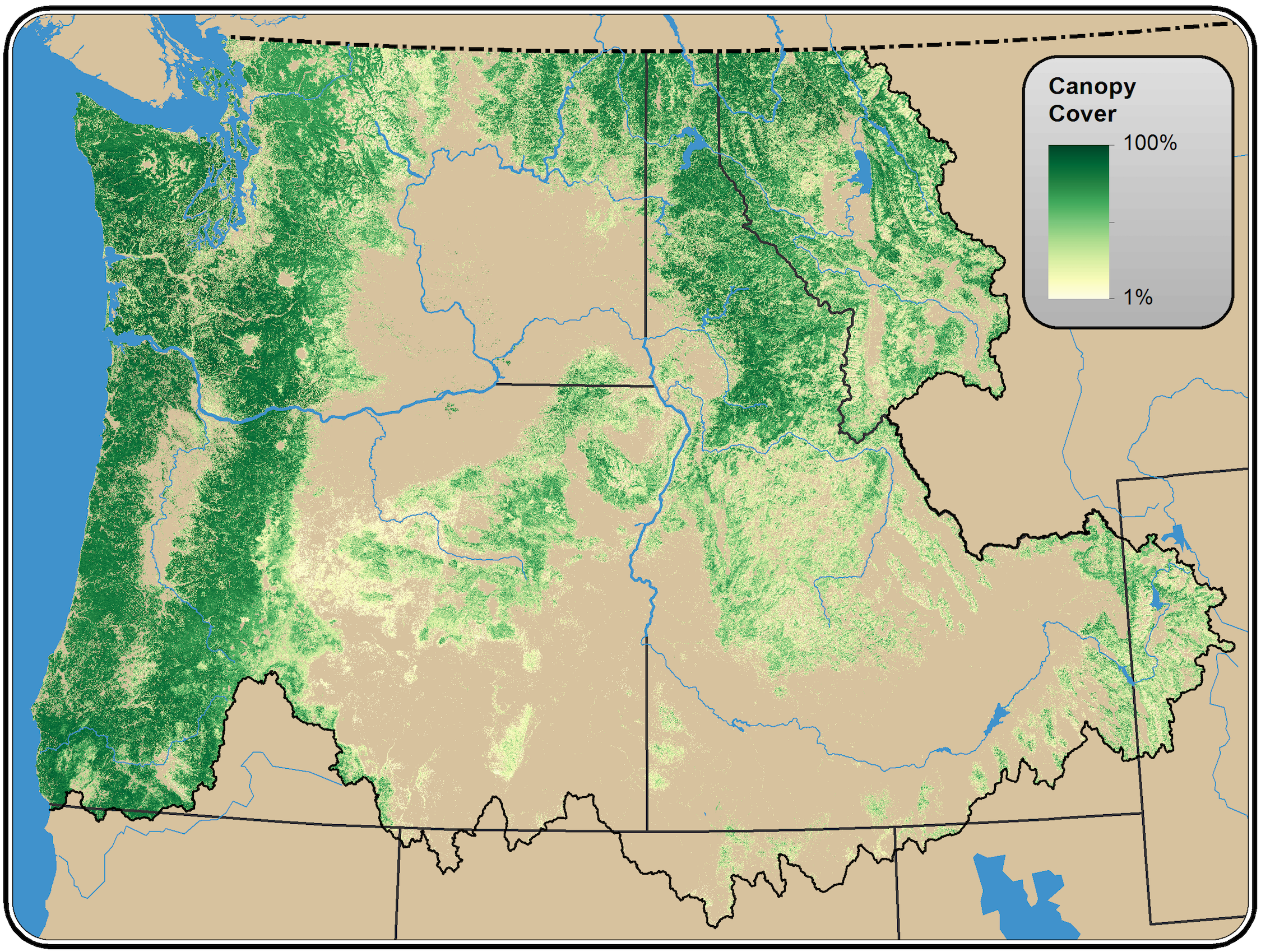

Supplement: S2 Fig — (TIFF) [file pone.0205156.s002.tiff]

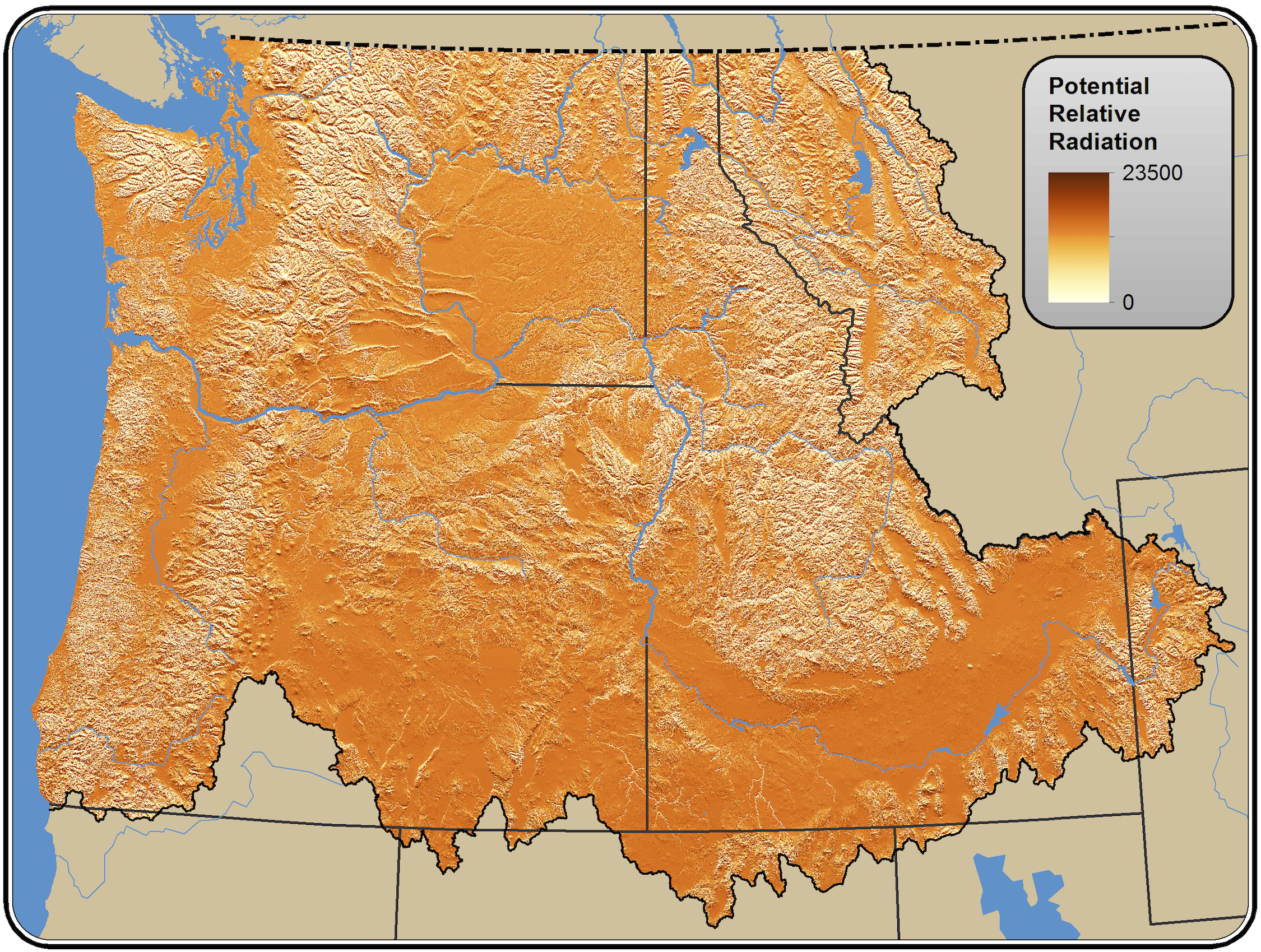

Supplement: S3 Fig — (TIFF) [file pone.0205156.s003.tiff]

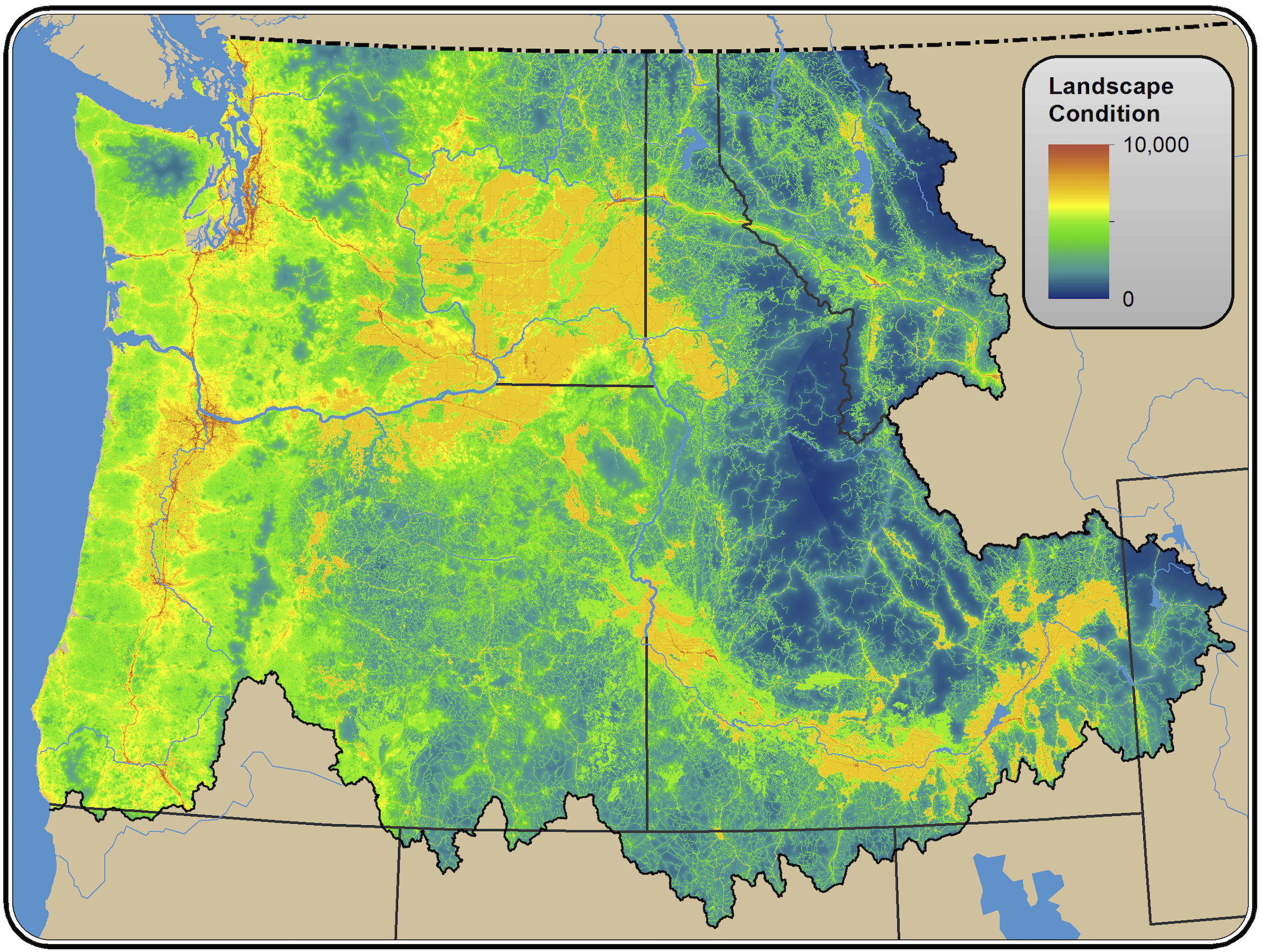

Supplement: S4 Fig — (TIFF) [file pone.0205156.s004.tiff]

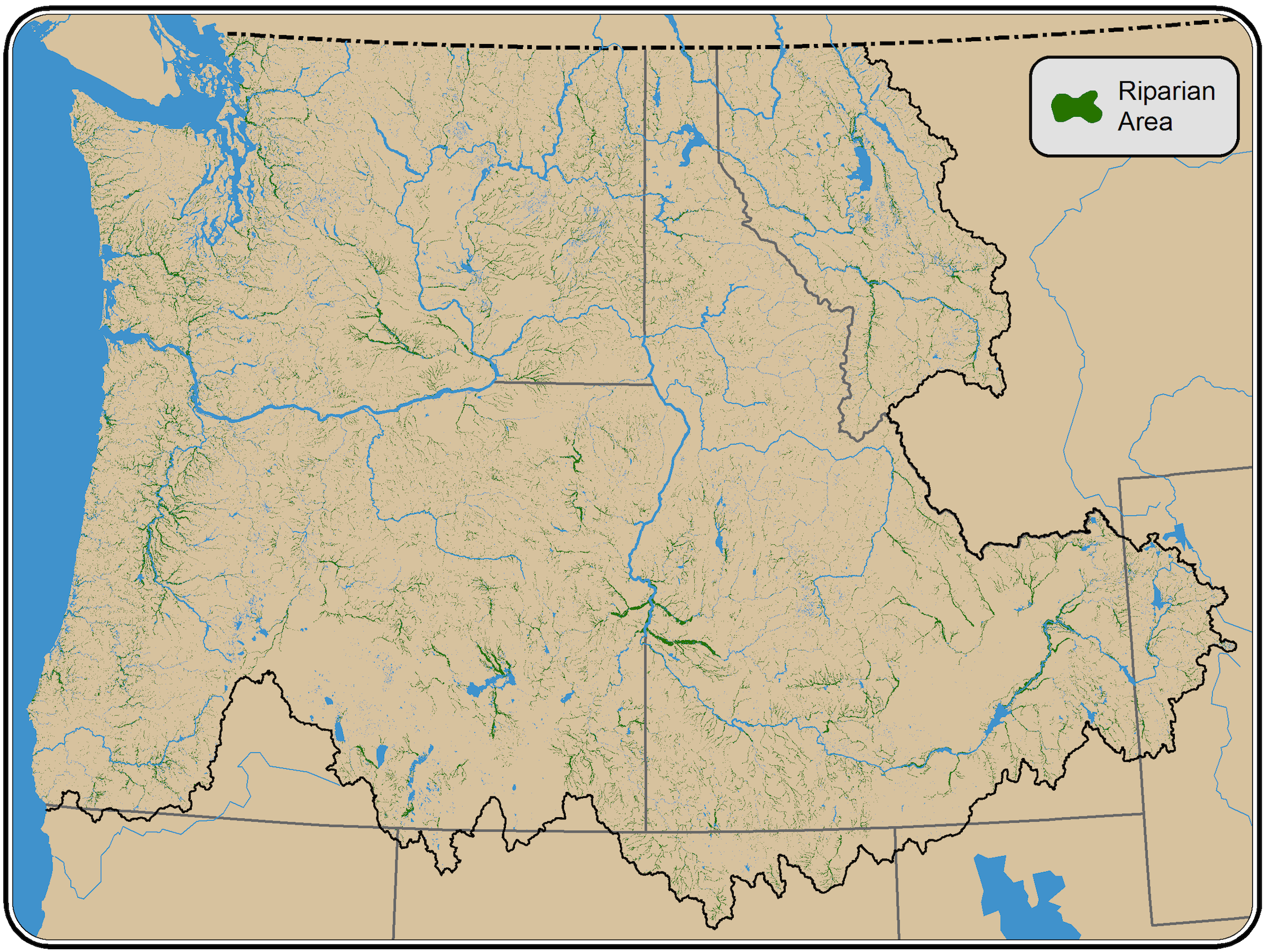

Supplement: S5 Fig — (TIFF) [file pone.0205156.s005.tiff]

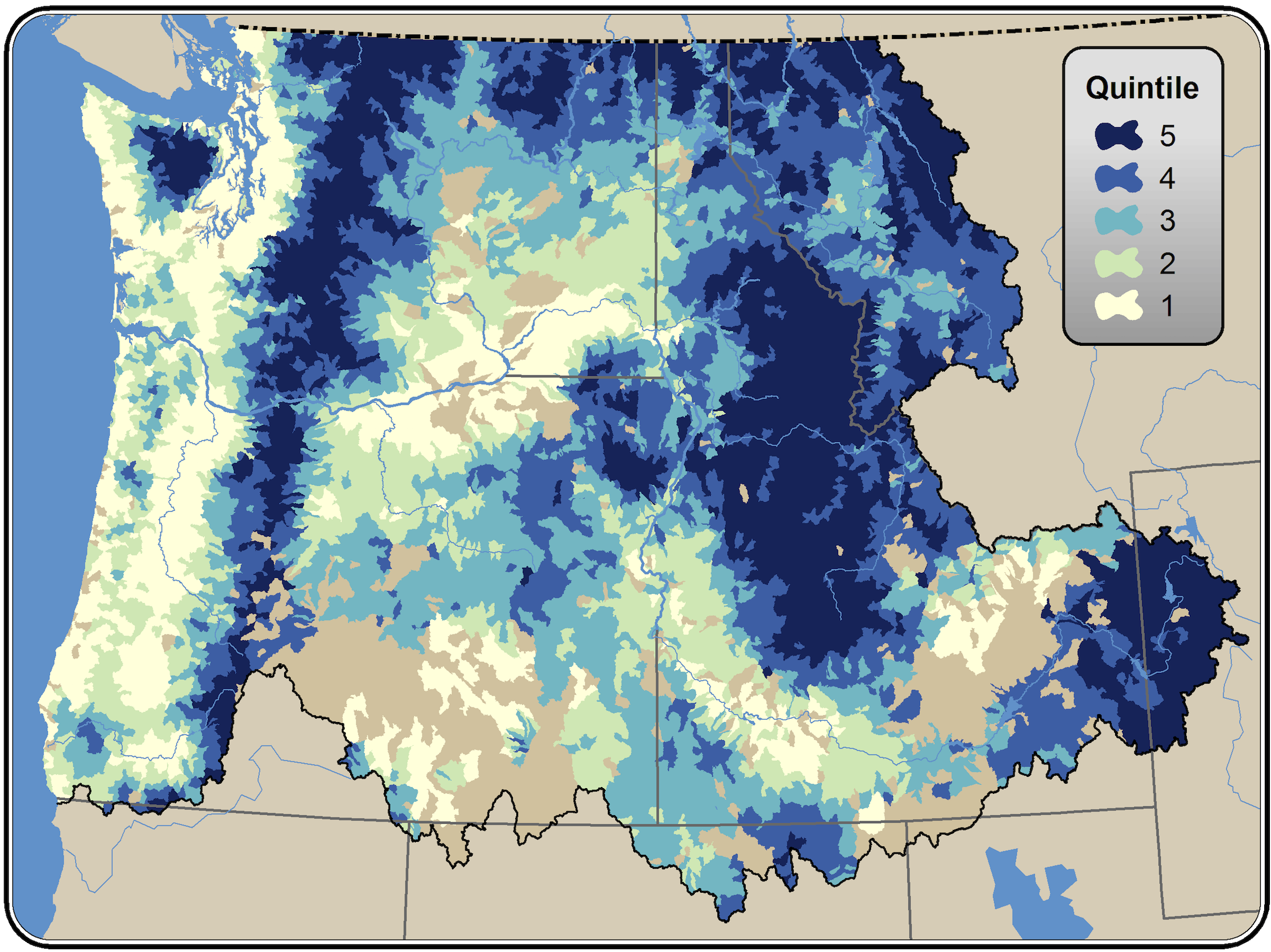

Supplement: S6 Fig — (TIFF) [file pone.0205156.s006.tiff]

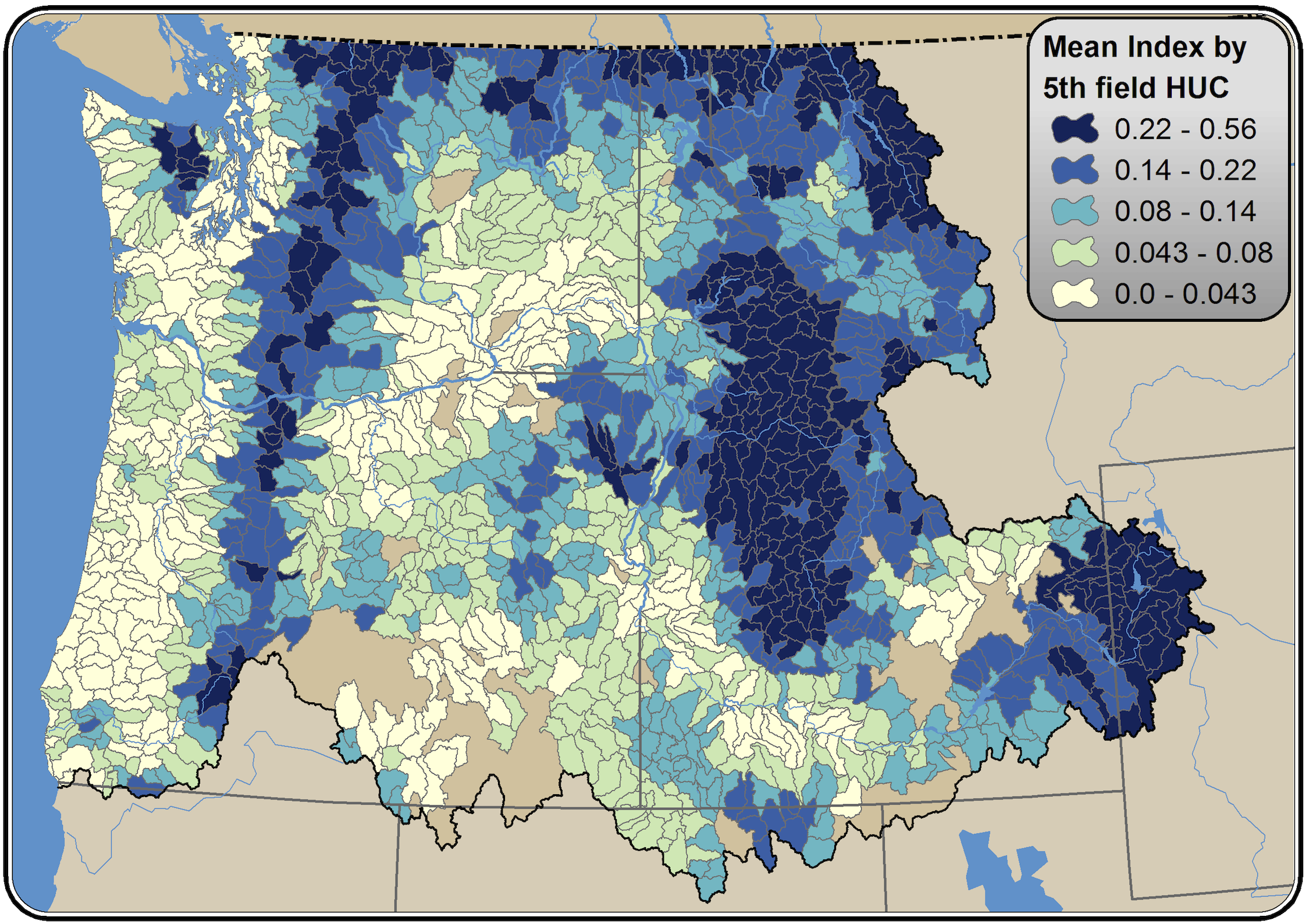

Supplement: S7 Fig — (TIFF) [file pone.0205156.s007.tiff]

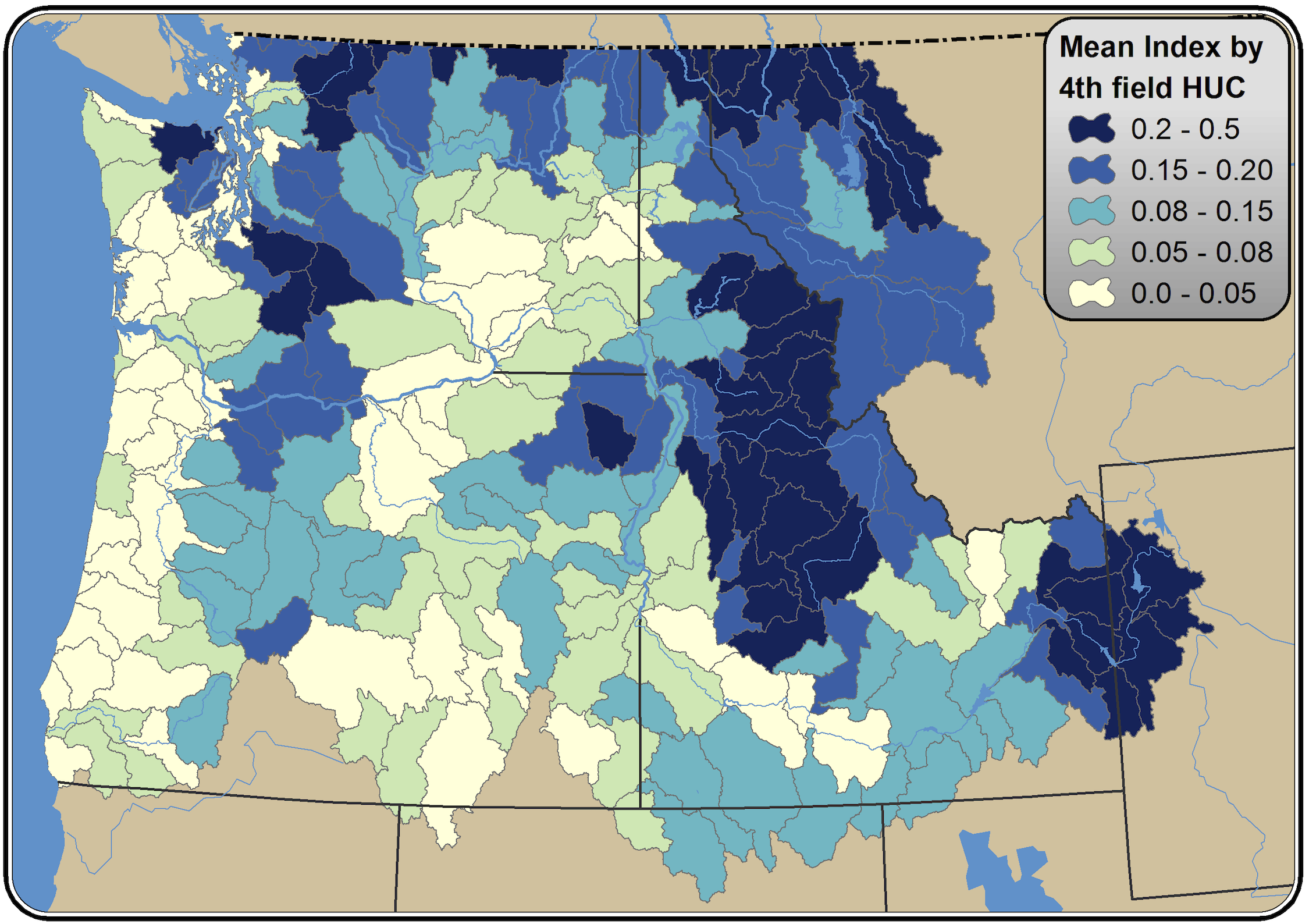

Supplement: S8 Fig — (TIFF) [file pone.0205156.s008.tiff]

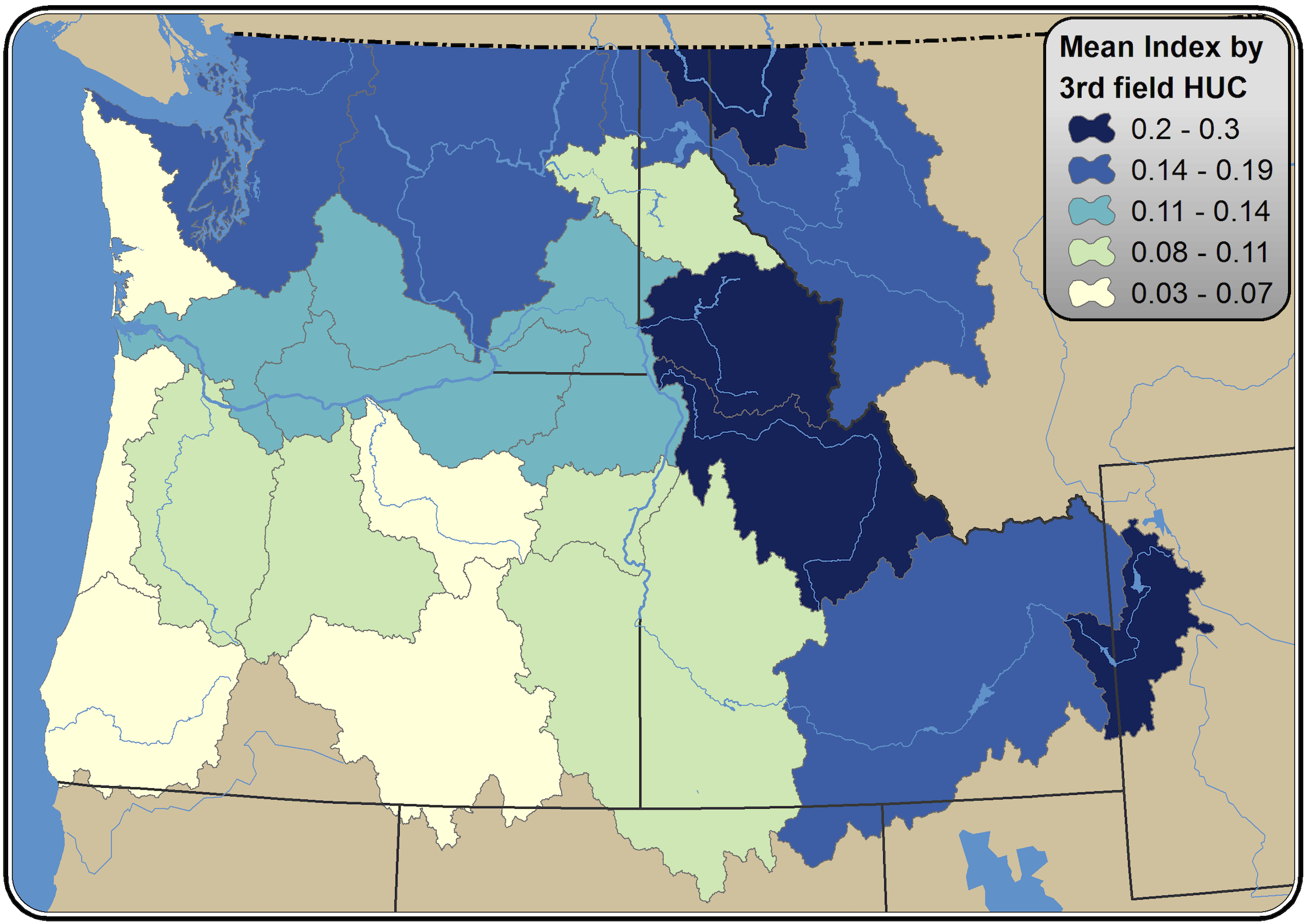

Supplement: S9 Fig — (TIFF) [file pone.0205156.s009.tiff]

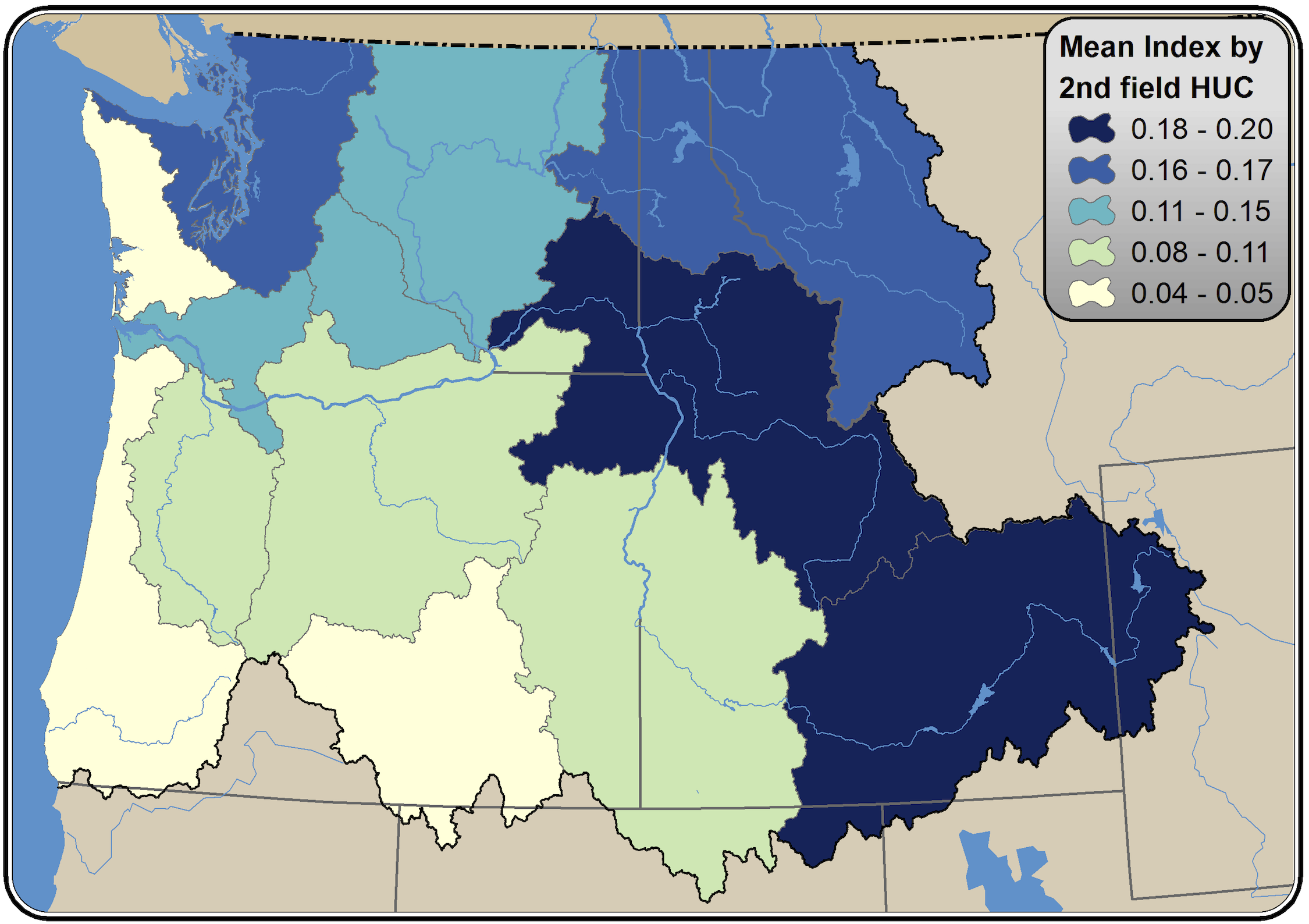

Supplement: S10 Fig — (TIFF) [file pone.0205156.s010.tiff]
